# Community physical activity environments and physical activity levels among older adults: a hierarchical analysis of environmental dimensions

**DOI:** 10.3389/fpubh.2026.1820476

**Published:** 2026-07-08

**Authors:** Huijun Yang, Xinjie Zhou, Qiyue Sun, Xu Li, Qiulu Chen

**Affiliations:** 1 School of Physical Education, Chongqing University of Posts and Telecommunications, Chongqing, China; 2 Chongqing Key Research Base of Humanities and Social Sciences Network Social Development Research Center, Chongqing, China; 3 Department of Physical Education, Shanghai Zhabei No. 8 Middle School, Shanghai, China; 4 Chengdu Sport University, Chengdu, China; 5 Collaborative Innovation Team for the Study and Interpretation of Xi Jinping’s Important Discourses on Cyberspace Governance, Chongqing University of Posts and Telecommunications, Chongqing, China; 6 School of Foreign Languages, Southwest University of Political Science and Law, Chongqing, China

**Keywords:** active aging, built and social environment, community environment, older adults, physical activity

## Abstract

**Background:**

Promoting physical activity among older adults is a major public health priority in aging societies. Although individual determinants of physical activity have been widely examined, increasing evidence suggests that community environmental conditions also shape behavioral participation. However, limited research has compared the relative associations of multiple community environmental dimensions with physical activity while accounting for demographic and lifestyle factors. This study examined the association between community physical activity environments and physical activity levels among older adults.

**Methods:**

A cross-sectional survey was conducted among 564 community-dwelling adults aged 60 years and above residing in nine urban districts. Physical activity levels were assessed using the Physical Activity Scale for the Elderly (PASE). Community physical activity environments were measured across four dimensions: natural environment, physical activity facilities, public services, and community physical activity culture. Hierarchical multiple regression analyses were performed to examine the associations between environmental indicators and physical activity levels after adjusting for demographic characteristics and lifestyle covariates.

**Results:**

The overall community physical activity environment showed a positive association with physical activity levels among older adults. The inclusion of environmental variables improved model explanatory power beyond demographic factors. Among the environmental dimensions, the natural environment and community physical activity culture showed comparatively stronger positive associations with physical activity levels. However, the high inter-correlations among environmental dimensions suggest that the coefficients for physical activity facilities and public services should be interpreted cautiously, as they may reflect shared variance or possible suppression effects.

**Conclusion:**

Supportive community physical activity environments are associated with higher physical activity levels among older adults, particularly in relation to favorable natural environmental conditions and socially supportive activity cultures. These findings suggest that public health strategies for active aging should not focus solely on facility provision, but should also enhance neighborhood environmental quality and foster community participation cultures. Because the study used cross-sectional self-reported data, the findings should be interpreted as associations rather than causal effects.

## 1 Introduction

Population aging has emerged as one of the most significant demographic transformations of the 21st century, creating substantial challenges for public health systems worldwide (1). Promoting healthy aging has therefore become a central priority in global health agendas (2), with regular physical activity widely recognized as a key behavioral strategy for maintaining functional capacity, preventing chronic diseases, and enhancing overall quality of life among older adults (3). Nevertheless, despite the well-documented benefits of physical activity, participation levels among older populations remain insufficient in many settings (4), highlighting the need to better understand the determinants of physical activity engagement in later life.

Early research on physical activity behavior primarily emphasized individual-level determinants, including health status, motivation, knowledge, and behavioral intentions (5). While these factors remain important, an increasing body of public health research has shifted toward ecological perspectives (6), which emphasize the role of environmental and contextual conditions in shaping behavioral outcomes (7). Ecological models suggest that individuals’ opportunities and motivations for engaging in physical activity are strongly influenced by the environments in which they live (8), including neighborhood infrastructure, accessibility of recreational resources, environmental safety, and community-level social norms (9). Such environmental influences may be particularly salient for older adults, who often rely more heavily on local community environments due to mobility limitations, safety concerns, and reduced spatial activity ranges.

Empirical studies conducted across diverse geographic contexts have consistently demonstrated positive associations between supportive neighborhood environments and physical activity participation among older adults (10). Features such as accessible green spaces, pedestrian-friendly neighborhoods, availability of recreational facilities, and supportive social environments have been shown to facilitate physical activity engagement (11). However, two issues remain insufficiently addressed. First, much of the existing literature has examined environmental indicators either in isolation or through aggregated environmental indices (12), which limits the ability to identify the relative contribution of specific environmental dimensions to physical activity participation (13). Second, comparatively limited research has incorporated lifestyle characteristics into the same analytical framework, even though these factors may confound the relationship between environmental conditions and physical activity outcomes (14). These limitations make it necessary to examine community environmental dimensions and lifestyle covariates within an integrated modeling framework.

In addition, community-level socio-cultural environmental factors remain under-examined. While built-environment indicators such as facility availability and infrastructure accessibility have received considerable research attention (11), the role of community participation culture, social encouragement, and collective behavioral norms has been less systematically examined (15). This omission is important because social environmental contexts may influence older adults’ physical activity through motivation, peer support, perceived norms, and opportunities for collective participation (16). Therefore, a multidimensional framework that includes natural, infrastructural, service-related, and socio-cultural environmental dimensions is required to better understand how community environments are associated with physical activity in later life (17).

In response to these gaps, the present study investigates the association between community physical activity environments and physical activity levels among community-dwelling older adults using a hierarchical modeling approach (18, 19). Specifically, this study aims to: first, examine whether the overall community physical activity environment is associated with older adults’ physical activity levels; second, compare the relative associations of four environmental dimensions, namely natural environment, physical activity facilities, public service provision, and community physical activity culture; and third, assess whether these associations remain after adjustment for demographic characteristics and lifestyle factors. By clarifying the relative contribution of different environmental dimensions, this study provides empirical evidence for community-based interventions and policy strategies designed to promote active and healthy aging (20, 21).

## 2 Literature framework

### 2.1 Ecological perspectives on physical activity among older adults

Ecological models of health behavior emphasize that behavioral outcomes cannot be adequately understood through individual-level characteristics alone (22); rather, they are embedded within broader contextual systems that include neighborhood environments (23), community infrastructure (24, 25), institutional support (26), and socio-cultural norms (27). Within this multi-level framework, environmental conditions influence physical activity not only by shaping the physical opportunities available for engagement but also by affecting perceived accessibility, safety, and social acceptability of activity participation (28). Consequently, the ecological perspective has become a dominant theoretical foundation in contemporary research examining behavioral health outcomes among aging populations (29).

For older adults in particular, environmental contexts may exert stronger influences than in younger populations (30) because age-related functional decline, mobility limitations, and heightened safety concerns often restrict the geographical range of daily activities (31). As a result, the immediate residential environment becomes a primary behavioral setting in which physical activity opportunities are either facilitated or constrained (32). Features such as the availability of accessible green spaces, pedestrian-friendly neighborhood design, well-maintained recreational facilities, and proximity of community-based activity programs can significantly reduce structural barriers to participation (33). At the same time, perceived environmental safety (34), neighborhood cohesion (35) (36), and community-level activity norms (37) can enhance behavioral motivation by fostering psychological comfort and social encouragement, both of which are critical for sustained activity engagement among older adults (38).

Existing empirical evidence generally supports the ecological assumption that supportive neighborhood environments are associated with higher levels of physical activity among older adults (39–41). However, this relationship should be understood as multidimensional rather than as the effect of a single environmental attribute. Natural environmental quality, facility availability, public service provision, and socio-cultural activity norms may jointly shape physical activity opportunities and motivations, but these dimensions are also likely to be conceptually related and empirically overlapping. Therefore, examining multiple environmental dimensions within a unified framework is necessary not only to identify their relative associations with physical activity, but also to avoid over-interpreting any single coefficient when shared variance exists among environmental indicators (42, 43).

In the present study, the ecological model of health behavior serves as the theoretical foundation for the research design. This model guided the study in three ways. First, it supported the inclusion of community environmental factors as contextual conditions associated with older adults’ physical activity. Second, it justified the multidimensional classification of the community physical activity environment into natural environment, physical activity facilities, public services, and community physical activity culture, thereby reflecting both physical and socio-cultural environmental influences. Third, it informed the hierarchical modeling strategy, in which demographic characteristics, environmental indicators, and lifestyle covariates were entered sequentially to examine whether community environmental associations remained after accounting for individual-level factors. Therefore, the ecological model provided the theoretical logic for linking environmental context, individual characteristics, and physical activity outcomes in this study.

### 2.2 Community physical activity environments and behavioral engagement

The concept of the community physical activity environment extends well beyond the mere presence of physical infrastructure and encompasses a multidimensional set of environmental conditions that collectively shape behavioral opportunities and motivations (44). Within public health and behavioral science literature (45), community physical activity environments are increasingly conceptualized as integrated systems (46) comprising natural environmental quality, accessibility and adequacy of physical activity facilities, availability of organized public services, and the broader socio-cultural atmosphere surrounding physical activity participation (47). Although these dimensions may influence behavioral engagement through distinct mechanisms, they are also likely to be interrelated in real community settings. Therefore, their independent associations should be interpreted with attention to potential conceptual overlap and shared explanatory variance.

Natural environmental quality constitutes a foundational component of community physical activity environments (48). Features such as clean air, green and open spaces, favorable microclimates, and esthetically appealing surroundings have been shown to enhance psychological well-being, reduce perceived exertion, and increase intrinsic motivation for physical activity participation (49). For older adults, who may be particularly sensitive to environmental comfort and safety, favorable natural conditions can reduce perceived environmental barriers and increase willingness to engage in outdoor and leisure-time physical activity (50). Empirical studies have demonstrated that access to green spaces and natural landscapes is associated with higher levels of walking, recreational activity, and overall physical activity among aging populations (51), suggesting that natural environmental quality plays both a direct and indirect role in shaping behavioral engagement (52).

The accessibility and adequacy of physical activity facilities represent another critical environmental dimension (53). Proximity to well-maintained and age-appropriate facilities, such as walking paths, exercise equipment, and community sports venues, can substantially lower logistical and physical constraints associated with activity participation (54). From an ecological perspective, accessible facilities increase opportunity structures for behavior by reducing time costs, transportation barriers, and physical effort required to initiate participation (55). However, evidence suggests that facility availability alone may be insufficient to guarantee sustained behavioral engagement, particularly when facilities are perceived as overcrowded, poorly maintained, or inadequately aligned with the functional capacities and preferences of older adults (56).

Public service provision further complements infrastructural resources by creating structured and socially supported opportunities for physical activity engagement (57). Community-organized exercise programs, health promotion initiatives, and age-targeted physical activity interventions can enhance participation by providing guidance, social interaction, and routine behavioral cues (58). Such services are particularly important for older adults who may lack confidence in independent exercise participation or require additional support to initiate and maintain regular activity (59). Studies have indicated that participation in organized community programs is associated with higher adherence to physical activity routines and improved long-term behavioral maintenance among older populations (60).

Beyond physical and organizational resources, the socio-cultural dimension of community physical activity environments plays a pivotal role in shaping behavioral engagement (61). CUL reflects social encouragement, collective participation norms, and supportive community attitudes, and it can influence behavior by shaping motivational climates and reinforcing behavioral intentions (62). Social environmental components, including perceived social cohesion, visibility of active peers, and normative expectations surrounding physical activity, may exert particularly strong influences among older adults (63), whose behavioral decisions are often closely tied to social belonging and community identity (64). Emerging evidence suggests that these social and cultural factors may, in some contexts, exert stronger effects on physical activity participation than purely infrastructural characteristics, underscoring the importance of considering community environments as socio-ecological systems rather than solely physical settings (65).

Taken together, existing research highlights the need for a multidimensional conceptualization of community physical activity environments that integrates natural, infrastructural, service-related, and socio-cultural elements (9). At the same time, these environmental dimensions should not be regarded as completely independent from one another. In actual community contexts, facility provision, public services, environmental quality, and activity culture may coexist and reinforce one another, leading to shared variance across environmental indicators. Therefore, a multidimensional analytical framework can help identify the relative associations of different environmental dimensions, but the coefficients of highly related dimensions should be interpreted cautiously rather than as isolated effects (66).

### 2.3 Behavioral and lifestyle determinants as confounding influences

Although environmental characteristics are increasingly recognized as key determinants of physical activity behavior (67), a growing body of public health research emphasizes that behavioral and lifestyle factors also exert substantial influences on participation patterns (68). Health-related behaviors such as smoking, alcohol consumption, dietary practices, and habitual activity routines are often embedded within broader lifestyle orientations that reflect individuals’ health awareness, behavioral preferences, and long-term behavioral regulation capacities (69). These lifestyle orientations can shape both the propensity to engage in physical activity and the responsiveness to environmental opportunities for participation (70). Consequently, failing to account for behavioral and lifestyle characteristics may lead to an overestimation or misinterpretation of environmental associations in empirical analyses (71).

Previous studies have consistently shown that individuals with healthier behavioral profiles, such as lower tobacco and alcohol consumption (72), balanced dietary habits (73), and regular engagement in leisure-time activity (74) (75), are more likely to participate in physical activity programs and maintain sustained activity routines (76). These behavioral patterns are frequently associated with higher levels of health consciousness, stronger intrinsic motivation for preventive health behaviors, and greater perceived self-efficacy in managing physical health (77). In contrast, individuals exhibiting unhealthy lifestyle patterns may encounter both physiological and psychological barriers to physical activity participation (78), including lower physical fitness, reduced behavioral readiness, and weaker health-related motivation (79). As a result, lifestyle characteristics may independently influence physical activity outcomes while simultaneously interacting with environmental conditions to shape behavioral responses (32).

Importantly, behavioral and lifestyle factors may also act as confounding influences in the association between environmental conditions and physical activity participation (32). For example, individuals with healthier lifestyles may be more likely to reside in neighborhoods with better environmental conditions or may be more inclined to utilize available environmental resources, leading to potential selection effects (80). Conversely, individuals with less favorable lifestyle patterns may underutilize existing environmental opportunities even when supportive facilities and services are available (81). Without appropriate statistical adjustment for these behavioral differences, observed environmental associations may partially reflect underlying lifestyle heterogeneity rather than independent environmental associations (82).

To address these analytical challenges, contemporary ecological and behavioral epidemiology research increasingly advocates the integration of demographic, environmental, and behavioral determinants within unified analytical frameworks (83). Incorporating behavioral covariates into regression models allows researchers to estimate the association between environmental conditions and physical activity while controlling for lifestyle-related influences, thereby providing a more cautious assessment of contextual environmental effects (84). In the present study, smoking frequency, alcohol consumption frequency, high-fat food consumption frequency, and physical activity frequency were considered lifestyle covariates. Among these variables, physical activity frequency should be distinguished from the overall physical activity outcome measured by the Physical Activity Scale for the Elderly, as it reflects the general frequency of participation rather than the multi-domain weighted activity score. Such an integrated analytical approach is particularly important in studies involving older adults, where lifestyle heterogeneity and accumulated health behaviors across the life course may substantially influence physical activity participation (85). By accounting for behavioral and lifestyle determinants, empirical analyses can more appropriately evaluate environmental associations with physical activity engagement and generate evidence for community-level public health interventions (13).

### 2.4 Research gaps and study contribution

Despite growing interest in environmental determinants of physical activity, several limitations remain in the existing literature (86). First, many studies have relied on aggregated environmental indices without sufficiently examining the relative associations of individual environmental dimensions, limiting the ability to identify which components of the community environment are more closely related to physical activity participation (12). Second, although natural environment, facility provision, public services, and community activity culture may represent distinct conceptual dimensions, they are often interrelated in real community settings. This raises the need to consider potential conceptual overlap and shared variance when interpreting dimension-specific coefficients. Third, relatively few studies have examined environmental factors alongside lifestyle covariates in hierarchical modeling frameworks, making it difficult to distinguish environmental associations from behavioral and lifestyle-related influences (87). In addition, evidence from rapidly urbanizing regions remains comparatively limited, despite the potential for substantial environmental heterogeneity across urban communities (88).

To address these gaps, the present study investigates the association between community physical activity environments and physical activity levels among older adults using a hierarchical modeling approach that sequentially incorporates demographic controls, environmental indicators, and lifestyle covariates (89). Specifically, this study aims to examine: first, whether the overall community physical activity environment is associated with older adults’ physical activity levels; second, how different environmental dimensions, including natural environment, physical activity facilities, public service provision, and community physical activity culture, are associated with physical activity levels; and third, whether these associations remain after adjustment for demographic and lifestyle factors. By examining both overall and dimension-specific environmental associations, this study contributes to a more cautious and nuanced understanding of how community environments are related to physical activity participation in later life, while also providing evidence for community-based active aging interventions (90, 91).

## 3 Methods

### 3.1 Study design

This study employed a cross-sectional survey design to examine the association between community physical activity environments and physical activity levels among older adults. Guided by ecological models of health behavior, which emphasize that physical activity is shaped by the interaction between individual characteristics and environmental contexts, the study investigated whether community-level environmental characteristics were associated with older adults’ physical activity after accounting for demographic and lifestyle factors. The research framework incorporated environmental indicators, demographic controls, and lifestyle variables to evaluate both unadjusted and adjusted associations between environmental characteristics and physical activity outcomes.

**Figure 1:**
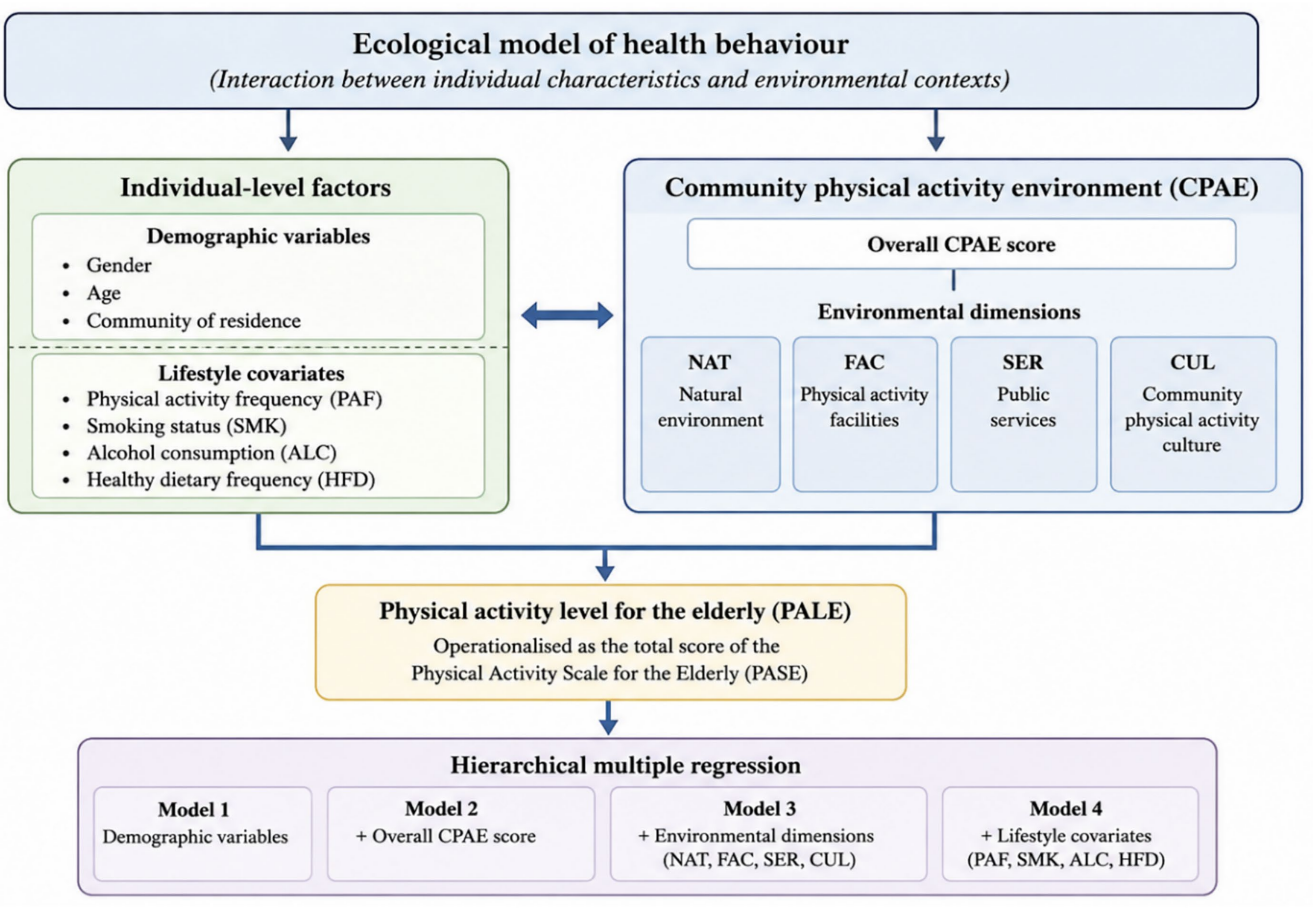
Conceptual framework of the study.

Figure 1 presents the conceptual framework of the study. The ecological model of health behavior guided the selection of variables and the hierarchical modeling strategy. Community physical activity environments were represented by both an overall environmental score and four environmental dimensions, while demographic and lifestyle factors were included as individual-level covariates. Physical activity level for the older adults (PALE), operationalized using the total score of the Physical Activity Scale for the Elderly (PASE), was treated as the outcome variable.

### 3.2 Research hypotheses

Based on the ecological model of health behavior and prior empirical evidence, the present study proposes the following hypotheses:

*H1*: The overall community physical activity environment is positively associated with physical activity levels among older adults.

*H2*: The four dimensions of the community physical activity environment, namely natural environment, physical activity facilities, public services, and community physical activity culture, are positively associated with physical activity levels among older adults.

*H3*: Among the environmental dimensions, natural environment and community physical activity culture are expected to show comparatively stronger associations with physical activity levels.

*H4*: The association between community physical activity environments and physical activity levels remains significant after adjustment for demographic and lifestyle factors.

### 3.3 Participants and sampling procedure

Participants were community-dwelling older adults aged 60 years and above residing in nine urban districts. Eligibility criteria required that respondents were capable of independent daily living, possessed adequate communication ability, and were able to understand and complete the questionnaire independently or with structured assistance from trained investigators. Individuals with severe mobility limitations or cognitive impairments that prevented questionnaire completion were excluded.

Given the limited feasibility of large-scale online surveys among older populations, a field-based data collection strategy was adopted. Participants were recruited through on-site random intercept sampling conducted in community public squares, neighborhood open spaces, and residential areas. Trained investigators approached potential respondents and explained the study objectives, voluntary participation procedures, and confidentiality assurances. For participants who required assistance, investigators provided structured guidance to ensure accurate questionnaire completion while maintaining respondent autonomy.

A total of 597 questionnaires were distributed, of which 564 valid questionnaires were returned, yielding a response rate of 94.5%. The achieved sample size exceeded commonly recommended thresholds for multivariate regression analyses, thereby ensuring adequate statistical power for the detection of medium-sized effects.

### 3.4 Ethical considerations

The study protocol was reviewed and approved by the relevant institutional ethics committee (Approval No. 202506200102386). All participants provided informed consent prior to participation. Respondents were informed that participation was voluntary, that they could withdraw at any time without consequence, and that all responses would be used exclusively for academic research purposes and reported only in aggregated form. No personally identifiable information was collected, thereby ensuring participant anonymity and data confidentiality.

Because both environmental perceptions and physical activity-related variables were collected through self-reported questionnaires at the same time point, procedural steps were taken to minimize potential common method and same-source bias. These included anonymous responses, confidentiality assurances, standardized instructions, and the separation of different questionnaire sections. Respondents were also informed that there were no right or wrong answers and were encouraged to answer according to their actual experiences.

### 3.5 Measures

#### 3.5.1 Physical activity level

Physical activity level for the older adults (PALE) was assessed using the Physical Activity Scale for the Elderly (PASE), a standardized questionnaire specifically developed to measure physical activity among older adults. The PASE assesses self-reported physical activity performed during the previous 7 days and covers three main domains: leisure-time activity, household activity, and occupational or work-related activity. It records information on the frequency, duration, and type of activities performed. Following the standard scoring procedure, responses were converted into weighted item scores and summed to generate a total PASE score. In the present study, this total PASE score was used to operationalize PALE as a continuous outcome variable, with higher scores indicating higher overall physical activity levels. The Chinese version of the PASE has demonstrated satisfactory reliability and construct validity in prior studies and was therefore adopted without modification in the present research. To avoid conceptual ambiguity, PASE refers to the measurement instrument, whereas PALE refers to the outcome variable derived from the total PASE score.

#### 3.5.2 Community physical activity environment

The community physical activity environment was measured using a structured questionnaire developed based on prior community sports environment evaluation frameworks. The instrument comprised four primary environmental dimensions:

*Community natural environment for physical activity (NAT)*, assessing environmental quality, green space availability, and environmental suitability for physical activity.

*FAC*, evaluating accessibility, availability, and adequacy of sports and recreational facilities.

*Community physical activity public services (SER)*, assessing organizational support, program availability, and service accessibility.

*CUL*, reflecting social norms, community engagement, and cultural support for physical activity participation.

Responses were aggregated to produce both dimension-specific scores and an overall community physical activity environment score (CPAE), with higher values indicating more supportive environments. Because these four environmental dimensions may be conceptually related in real community settings, both the overall CPAE score and the dimension-specific scores were retained for analysis.

#### 3.5.3 Lifestyle and demographic variables

Demographic control variables included gender, age group, and community of residence. Lifestyle covariates included smoking frequency (SMK), alcohol consumption frequency (ALC), frequency of high-fat food consumption (HFD), and physical activity participation frequency (PAF). PAF was measured as a single-item behavioral variable reflecting respondents’ general weekly frequency of physical activity participation. It should be distinguished from PALE: PALE was derived from the multi-domain weighted PASE total score and served as the dependent variable, whereas PAF was included as a lifestyle covariate to adjust for habitual activity frequency. These variables were included to control for behavioral factors that may be associated with physical activity participation independently of environmental characteristics.

### 3.6 Reliability and validity assessment

The internal consistency reliability of the community physical activity environment scale was evaluated using Cronbach’s alpha coefficient, which yielded a value of 0.898, indicating excellent reliability. Construct validity was assessed using the Kaiser–Meyer–Olkin (KMO) measure of sampling adequacy, which produced a value of 0.916, demonstrating strong suitability for factor-based structural validation and supporting the adequacy of the measurement structure. As the PASE is an internationally validated instrument with established psychometric properties and was adopted without modification, additional scale development procedures for the PALE outcome were not conducted.

### 3.7 Statistical analysis

#### 3.7.1 Preliminary analyses

Descriptive statistics, including means, standard deviations, and distributional characteristics, were calculated to summarize sample characteristics. Pearson correlation analyses were conducted to examine bivariate associations among the main study variables and to provide preliminary evidence of relationships between environmental indicators and physical activity levels.

Regression assumptions were evaluated prior to model estimation. Linearity was assessed through visual inspection of scatterplots, which indicated an approximately linear relationship between environmental indicators and PALE. Multicollinearity was examined using variance inflation factors (VIFs), and residual diagnostics were performed using normal probability plots and scatterplots of standardized predicted values (ZPRED) against standardized residuals (ZRESID) to assess homoscedasticity and normality assumptions. Given the high correlations expected among environmental dimensions, both bivariate correlations and VIF values were considered when interpreting the regression coefficients. Particular caution was applied to the interpretation of FAC and SER coefficients because their associations may overlap with other environmental dimensions.

#### 3.7.2 Regression modeling

Hierarchical multiple linear regression analyses were performed to examine the associations between environmental characteristics and PALE.

Model 1 (baseline model) included demographic control variables (gender, age, and community) to establish baseline explanatory power.

Model 2 (main-effect model) introduced the overall CPAE score to examine the association between the general community physical activity environment and PALE.

Model 3 (dimension-specific model) replaced the overall environmental score with the four environmental dimensions (NAT, FAC, SER, and CUL) to examine their relative associations with PALE. Given the conceptual and empirical overlap among environmental dimensions, the coefficients in this model were interpreted as unique associations after mutual adjustment rather than as isolated total effects.

Model 4 (adjusted model) further incorporated lifestyle covariates (SMK, ALC, HFD, and PAF) to examine whether the association between environmental characteristics and PALE remained after controlling for behavioral differences. In this model, PAF was treated as a lifestyle covariate reflecting general activity frequency and was distinguished from PALE, which was derived from the total PASE score.

All analyses were conducted using SPSS statistical software, and statistical significance was set at *p* < 0.05.

## 4 Results

### 4.1 Participant characteristics and descriptive statistics

A total of 564 community-dwelling older adults were included in the present analysis. As shown in Table 1, the sample comprised 291 men (51.6%) and 273 women (48.4%). Participants were predominantly aged between 65 and 69 years (33.2%), followed by those aged 60–64 years (25.9%) and 70–74 years (24.1%), while smaller proportions were aged 75–79 years (14.0%) and 80 years or above (2.8%). Respondents were drawn from multiple districts, with the largest proportions residing in Nan’an District (18.6%), Dadukou District (16.5%), and Beibei District (15.4%).

**Table 1:** Participant characteristics and descriptive statistics (*N* = 564).

Categorical variables			
Variable	Category	*n*	%
Gender	Male	291	51.6
	Female	273	48.4
Age group	60–64 years	146	25.9
	65–69 years	187	33.2
	70–74 years	136	24.1
	75–79 years	79	14
	≥80 years	16	2.8
COMM	Yuzhong district	33	5.9
	Yubei district	45	8
	Nan’an district	105	18.6
	Banan district	39	6.9
	Jiangbei district	57	10.1
	Shapingba district	60	10.6
	Jiulongpo district	45	8
	Dadukou district	93	16.5
	Beibei district	87	15.4

Continuous variables		
Variable	Mean	SD
PALE	93.05	53.58
CPAE	3.64	0.56
NAT	3.69	0.77
FAC	3.65	0.58
SER	3.38	0.67
CUL	2.47	0.44

Lifestyle variables			
Variable	Category	*n*	%
PAF	Never	109	19.3
	1-2 times/week	173	30.7
	3–6 times/week	118	20.9
	≥7 times/week	164	29.1
SMK	Never	307	54.4
	1–10 cigarettes/day	179	31.7
	11–19 cigarettes/day	63	11.2
	≥20 cigarettes/day	15	2.7
ALC	Never	218	38.7
	1-2 times/week	261	46.3
	3–6 times/week	59	10.5
	≥7 times/week	26	4.6
HFD	Never	132	23.4
	1-2 times/week	253	44.9
	3–6 times/week	137	24.3
	≥7 times/week	42	7.4

Values are presented as mean (SD) or *n* (%). COMM, community location; PALE, physical activity level for the older adults, operationalized as the total score of the Physical Activity Scale for the Elderly (PASE); CPAE, overall community physical activity environment score; NAT, natural environment for physical activity; FAC, physical activity facilities; SER, public services for physical activity; CUL, community physical activity culture; PAF, physical activity frequency, measured as self-reported weekly participation frequency; SMK, smoking frequency; ALC, alcohol consumption frequency; HFD, high-fat food consumption frequency.

With regard to the key study variables, the mean PALE score was 93.05 (SD = 53.58), indicating variability in the physical activity levels of the participants. As defined in the Methods section, PALE was operationalized using the total score of the Physical Activity Scale for the Elderly (PASE). The mean CPAE score was 3.64 (SD = 0.56). Among the environmental dimensions, NAT showed the highest mean score (*M* = 3.69, SD = 0.77), followed by FAC (*M* = 3.65, SD = 0.58) and SER (*M* = 3.38, SD = 0.67), whereas CUL showed a comparatively lower mean score (*M* = 2.47, SD = 0.44).

In terms of lifestyle characteristics, PAF reflected participants’ self-reported frequency of physical activity participation and should be distinguished from PALE, which was derived from the multi-domain PASE total score. Overall, 19.3% of participants reported never engaging in regular physical activity, while 30.7% reported participating one to two times per week and 29.1% reported engaging in physical activity seven times per week or more. More than half of the respondents reported never smoking (54.4%), and 38.7% reported never consuming alcohol. Regarding dietary behavior, 44.9% of participants reported consuming high-fat foods one to two times per week, whereas only 7.4% reported such consumption seven times per week or more. Collectively, these findings indicate variability in physical activity, perceived community environments, and lifestyle characteristics within the study population.

### 4.2 Bivariate correlation analysis

Pearson’s correlation analyses were conducted to examine the bivariate associations among the study variables (Table 2). PALE was positively correlated with the overall CPAE score (*r* = 0.393, *p* < 0.001). At the dimension level, PALE showed significant positive correlations with NAT (*r* = 0.383, *p* < 0.001), FAC (*r* = 0.238, *p* < 0.001), SER (*r* = 0.361, *p* < 0.001), and CUL (*r* = 0.504, *p* < 0.001), with the strongest bivariate association observed for CUL.

**Table 2:** Pearson correlations.

Variables	Statistic	PALE	CPAE	NAT	FAC	SER	CUL	PAF	SMK	ALC	HFD
PALE	Pearson correlation	1	0.393**	0.383**	0.238**	0.361**	0.504**	0.195**	−0.238**	−0.162**	−0.167**
	Sig. (2-tailed)		0.000	0.000	0.000	0.000	0.000	0.000	0.000	0.000	0.000
	*N*	564	564	564	564	564	564	564	564	564	564
CPAE	Pearson correlation	0.393**	1	0.864**	0.895**	0.903**	0.593**	0.158**	−0.020	0.050	0.010
	Sig. (2-tailed)	0.000		0.000	0.000	0.000	0.000	0.000	0.637	0.233	0.811
	*N*	564	564	564	564	564	564	564	564	564	564
NAT	Pearson correlation	0.383**	0.864**	1	0.721**	0.733**	0.402**	0.130**	0.015	0.055	−0.006
	Sig. (2-tailed)	0.000	0.000		0.000	0.000	0.000	0.002	0.716	0.188	0.896
	*N*	564	564	564	564	564	564	564	564	564	564
FAC	Pearson correlation	0.238**	0.895**	0.721**	1	0.674**	0.362**	0.186**	0.034	0.053	0.108*
	Sig. (2-tailed)	0.000	0.000	0.000		0.000	0.000	0.000	0.417	0.212	0.010
	*N*	564	564	564	564	564	564	564	564	564	564
SER	Pearson correlation	0.361**	0.903**	0.733**	0.674**	1	0.568**	0.065	−0.036	0.039	−0.062
	Sig. (2-tailed)	0.000	0.000	0.000	0.000		0.000	0.123	0.397	0.352	0.142
	*N*	564	564	564	564	564	564	564	564	564	564
CUL	Pearson correlation	0.504**	0.593**	0.402**	0.362**	0.568**	1	0.174**	−0.168**	−0.022	−0.064
	Sig. (2-tailed)	0.000	0.000	0.000	0.000	0.000		0.000	0.000	0.608	0.128
	*N*	564	564	564	564	564	564	564	564	564	564
PAF	Pearson correlation	0.195**	0.158**	0.130**	0.186**	0.065	0.174**	1	−0.088*	−0.111**	0.037
	Sig. (2-tailed)	0.000	0.000	0.002	0.000	0.123	0.000		0.036	0.008	0.382
	*N*	564	564	564	564	564	564	564	564	564	564
SMK	Pearson correlation	−0.238**	−0.020	0.015	0.034	−0.036	−0.168**	−0.088*	1	0.345**	0.072
	Sig. (2-tailed)	0.000	0.637	0.716	0.417	0.397	0.000	0.036		0.000	0.086
	*N*	564	564	564	564	564	564	564	564	564	564
ALC	Pearson correlation	−0.162**	0.050	0.055	0.053	0.039	−0.022	−0.111**	0.345**	1	0.115**
	Sig. (2-tailed)	0.000	0.233	0.188	0.212	0.352	0.608	0.008	0.000		0.006
	*N*	564	564	564	564	564	564	564	564	564	564
HFD	Pearson correlation	−0.167**	0.010	−0.006	0.108*	−0.062	−0.064	0.037	0.072	0.115**	1
	Sig. (2-tailed)	0.000	0.811	0.896	0.010	0.142	0.128	0.382	0.086	0.006	
	*N*	564	564	564	564	564	564	564	564	564	564

PALE, physical activity level for the older adults, operationalized as the total score of the Physical Activity Scale for the Elderly (PASE); CPAE, overall community physical activity environment score; NAT, natural environment for physical activity; FAC, physical activity facilities; SER, public services for physical activity; CUL, community physical activity culture; PAF, physical activity frequency, measured as self-reported weekly participation frequency; SMK, smoking frequency; ALC, alcohol consumption frequency; HFD, high-fat food consumption frequency. **p < 0.01; *p < 0.05.

With respect to lifestyle variables, PALE was positively associated with PAF (*r* = 0.195, *p* < 0.001), whereas negative correlations were observed with SMK (*r* = −0.238, *p* < 0.001), ALC (*r* = −0.162, *p* < 0.001), and HFD (*r* = −0.167, *p* < 0.001). PAF should be interpreted as self-reported physical activity frequency rather than the multi-domain PASE-derived PALE outcome. In addition, the environmental indicators were strongly inter-correlated, particularly between CPAE and NAT (*r* = 0.864), FAC (*r* = 0.895), and SER (*r* = 0.903) (all *p* < 0.001), indicating substantial shared variance across environmental dimensions. These high correlations suggest that the subsequent dimension-specific regression coefficients, especially those for FAC and SER, should be interpreted cautiously because they may reflect conceptual overlap or suppression effects rather than isolated environmental effects. Multicollinearity was therefore further assessed in the regression analyses using variance inflation factors.

### 4.3 Hierarchical multiple regression results

Multiple linear regression analyses were performed to examine the associations between community physical activity environment indicators and PALE. The model fit statistics are presented in Table 3, the overall model significance tests are summarized in Table 4, and the estimated regression coefficients are shown in Table 5.

**Table 3:** Model summary^a^.

Model	*R*	*R* square	Adjusted *R* square	Std. error of the estimate	Change statistics				
					*R* square change	*F* change	d*f*1	d*f*2	Sig. *F* change
1	0.482^b^	0.232	0.228	47.0728	0.232	56.484	3	560	0.000
2	0.534^c^	0.286	0.281	45.4483	0.053	41.749	1	559	0.000
3	0.611^d^	0.374	0.366	42.6672	0.141	31.403	4	556	0.000
4	0.590^e^	0.348	0.338	43.5832	0.062	13.216	4	555	0.000

a Dependent variable: PALE, physical activity level for the older adults, operationalized as the total PASE score.

b Predictors: (constant), age, gender, COMM.

c Predictors: (constant), age, gender, COMM, CPAE.

d Predictors: (constant), age, gender, COMM, FAC, CUL, NAT, SER.

e Predictors: (constant), age, gender, COMM, CPAE, PAF, HFD, ALC, SMK.

**Table 4:** ANOVA^a^.

Model		Sum of squares	d*f*	Mean square	*F*	Sig.
1	Regression	375479.357	3	125159.786	56.484	0.000^b^
	Residual	1240873.031	560	2215.845		
	Total	1616352.387	563			
2	Regression	461713.158	4	115428.290	55.883	0.000^c^
	Residual	1154639.229	559	2065.544		
	Total	1616352.387	563			
3	Regression	604158.134	7	86308.305	47.409	0.000^d^
	Residual	1012194.253	556	1820.493		
	Total	1616352.387	563			
4	Regression	562131.642	8	70266.455	36.992	0.000^e^
	Residual	1054220.745	555	1899.497		
	Total	1616352.387	563			

a Dependent variable: PALE, physical activity level for the older adults, operationalized as the total PASE score.

b Predictors: (constant), age, gender, COMM.

c Predictors: (constant), age, gender, COMM, CPAE.

d Predictors: (constant), age, gender, COMM, FAC, CUL, NAT, SER.

e Predictors: (constant), age, gender, COMM, CPAE, PAF, HFD, ALC, SMK.

**Table 5:** Coefficients^a^.

Model		Unstandardized coefficients		Standardized coefficients	*t*	Sig.	Collinearity statistics	
		*B*	Std. error	Beta			Tolerance	VIF
1	(Constant)	13.666	8.330		1.641	0.101		
	COMM	8.904	0.782	0.428	11.393	0.000	0.970	1.031
	Gender	9.232	3.973	0.086	2.324	0.020	0.997	1.003
	Age	7.259	1.841	0.148	3.943	0.000	0.970	1.031
2	(Constant)	−62.869	14.317		−4.391	0.000		
	COMM	6.858	0.818	0.330	8.380	0.000	0.825	1.213
	Gender	9.750	3.837	0.091	2.541	0.011	0.996	1.004
	Age	6.901	1.778	0.141	3.880	0.000	0.969	1.032
	CPAE	24.111	3.732	0.252	6.461	0.000	0.842	1.188
3	(Constant)	−83.671	14.463		−5.785	0.000		
	COMM	5.388	0.803	0.259	6.712	0.000	0.755	1.324
	Gender	5.845	3.638	0.055	1.607	0.109	0.976	1.024
	Age	5.455	1.685	0.111	3.238	0.001	0.952	1.051
	NAT	16.651	3.943	0.239	4.223	0.000	0.352	2.842
	FAC	−5.603	4.781	−0.061	−1.172	0.242	0.420	2.378
	SER	−8.385	4.686	−0.104	−1.789	0.074	0.332	3.010
	CUL	45.859	5.136	0.375	8.930	0.000	0.640	1.562
4	(Constant)	−25.318	16.168		−1.566	0.118		
	COMM	5.939	0.815	0.286	7.286	0.000	0.764	1.308
	Gender	0.970	3.993	0.009	0.243	0.808	0.846	1.182
	Age	7.488	1.729	0.153	4.332	0.000	0.943	1.060
	CPAE	23.534	3.668	0.246	6.416	0.000	0.801	1.249
	PAF	6.684	1.713	0.137	3.903	0.000	0.950	1.053
	SMK	−10.234	2.641	−0.150	−3.875	0.000	0.781	1.281
	ALC	−5.655	2.505	−0.084	−2.257	0.024	0.839	1.192
	HFD	−4.319	2.216	−0.070	−1.949	0.052	0.915	1.093

a Dependent variable: PALE, physical activity level for the older adults, operationalized as the total PASE score.

#### 4.3.1 Model fit statistics

The hierarchical multiple regression results are summarized in Tables 3–5. As shown in Table 3, the baseline model including demographic control variables only (gender, age, and community) accounted for 23.2% of the variance in PALE (*R*^2^ = 0.232), indicating that demographic characteristics explained a meaningful proportion of variability in physical activity levels among older adults.

When the overall CPAE score was introduced in Model 2, the explanatory power increased to 28.6% (*R*^2^ = 0.286), with an *R*^2^ change of 0.053. This suggests that the overall community physical activity environment was associated with additional variation in PALE beyond demographic characteristics.

In Model 3, the overall environmental score was replaced by the four environmental dimensions (NAT, FAC, SER, and CUL). The explained variance increased to 37.4% (*R*^2^ = 0.374; Δ*R*^2^ = 0.141), suggesting that the dimension-specific environmental indicators collectively provided greater explanatory information than the demographic variables alone. However, because the environmental dimensions were strongly inter-correlated, the model fit improvement should be interpreted together with the coefficient estimates and collinearity diagnostics rather than as evidence that each dimension had an independent isolated effect.

Model 4 introduced lifestyle covariates (PAF, SMK, ALC, and HFD) together with the overall CPAE score. This model explained 34.8% of the variance in PALE (*R*^2^ = 0.348), indicating that the association between the overall community physical activity environment and PALE remained evident after adjustment for demographic and lifestyle characteristics. Because Model 4 used the overall CPAE score rather than the four separate environmental dimensions, it should be interpreted as an adjusted overall-environment model rather than a direct extension of the dimension-specific model.

#### 4.3.2 Overall model significance

The ANOVA results presented in Table 4 show that all four regression models were statistically significant at the 0.001 level, indicating that the sets of predictors included at each modeling stage jointly explained a significant proportion of the variance in PALE.

Specifically, the baseline model including only demographic control variables was statistically significant (Model 1: *F* (3, 560) = 56.48, *p* < 0.001), with a regression sum of squares of 375479.36. After the inclusion of the overall community physical activity environment indicator, Model 2 also remained statistically significant (*F* (4, 559) = 55.88, *p* < 0.001), with an increased regression sum of squares of 461713.16. This indicates that the overall CPAE score provided additional explanatory information beyond demographic characteristics.

When the environmental construct was represented by four specific environmental dimensions, Model 3 showed strong overall significance (*F* (7, 556) = 47.41, *p* < 0.001), and the regression sum of squares further increased to 604158.13. This suggests that the dimension-specific environmental indicators collectively explained additional variation in PALE. However, given the high inter-correlations among the environmental dimensions, the overall significance of Model 3 should be interpreted together with the coefficient estimates and collinearity diagnostics.

Model 4, which included the overall CPAE score and lifestyle covariates, was also statistically significant (*F* (8, 555) = 36.99, *p* < 0.001). This indicates that demographic characteristics, the overall community physical activity environment, and lifestyle variables jointly contributed to explaining variability in PALE. Because Model 4 used the overall CPAE score rather than the four separate environmental dimensions, it should be interpreted as an adjusted overall-environment model rather than as a direct continuation of the dimension-specific model.

Overall, the ANOVA results indicate that the regression specifications were statistically significant across modeling stages. These findings support further interpretation of the regression coefficients, while recognizing that dimension-specific coefficients should be interpreted cautiously in light of the shared variance among environmental indicators.

#### 4.3.3 Regression coefficients

The estimated regression coefficients presented in Table 5 provide further information on the associations of demographic, environmental, and lifestyle variables with PALE.

In the baseline model (Model 1), community location (COMM) was positively associated with PALE (*β* = 0.428, *p* < 0.001), indicating substantial between-community differences in physical activity levels. Age was also positively associated with PALE (*β* = 0.148, *p* < 0.001), while gender showed a modest but statistically significant association (*β* = 0.086, *p* = 0.020). These results suggest that demographic characteristics accounted for part of the variation in PALE before the inclusion of environmental indicators.

After the introduction of the overall environmental indicator in Model 2, CPAE was positively associated with PALE (*β* = 0.252, *p* < 0.001), indicating that more supportive community physical activity environments were related to higher physical activity levels among older adults. The coefficient for community location decreased from *β* = 0.428 to *β* = 0.330, suggesting that part of the between-community variation in PALE may be related to differences in perceived community environmental conditions. Age and gender remained statistically significant in this model.

When the environmental construct was represented by four specific dimensions in Model 3, NAT (*β* = 0.239, *p* < 0.001) and CUL (*β* = 0.375, *p* < 0.001) showed significant positive associations with PALE. These results suggest that favorable natural environmental conditions and supportive community physical activity culture were more consistently associated with higher physical activity levels. In contrast, FAC (*β* = −0.061, *p* = 0.242) and SER (*β* = −0.104, *p* = 0.074) were not statistically significant after simultaneous adjustment for the other environmental dimensions. Given the strong inter-correlations among environmental indicators reported in Table 2, these non-significant and negative coefficients should not be interpreted as evidence that facilities or public services are unimportant. Rather, they may reflect conceptual overlap, shared explanatory variance, or possible suppression effects when closely related environmental dimensions are entered into the same regression model.

In Model 4, which included the overall CPAE score and lifestyle covariates, CPAE remained positively associated with PALE (*β* = 0.246, *p* < 0.001). This indicates that the association between the overall community physical activity environment and PALE remained evident after adjustment for demographic characteristics and lifestyle variables. Among the lifestyle variables, PAF was positively associated with PALE (*β* = 0.137, *p* < 0.001). This result should be interpreted cautiously because PAF reflects self-reported physical activity frequency, whereas PALE was derived from the multi-domain weighted PASE total score. SMK (*β* = −0.150, *p* < 0.001) and ALC (*β* = −0.084, *p* = 0.024) were negatively associated with PALE, while HFD did not reach the conventional level of statistical significance (*β* = −0.070, *p* = 0.052).

Across the models, VIF values remained below conventional thresholds, suggesting that severe multicollinearity was not indicated by this diagnostic criterion. Nevertheless, the high bivariate correlations among environmental dimensions suggest that the dimension-specific coefficients, particularly those for FAC and SER, should be interpreted as unique associations after mutual adjustment rather than as isolated total environmental effects.

Taken together, the regression results indicate that the overall community physical activity environment was positively associated with PALE. At the dimension level, NAT and CUL showed more stable positive associations, whereas the coefficients for FAC and SER appeared less stable after adjustment for overlapping environmental dimensions.

## 5 Discussion

The present study provides empirical evidence that community-level environmental characteristics are significantly associated with physical activity levels among older adults (92), supporting the ecological perspective that behavioral outcomes are shaped not only by individual attributes but also by contextual environmental conditions (93). The findings show that the inclusion of environmental indicators improved the explanatory power of the regression models, suggesting that perceived community environments are relevant to older adults’ physical activity participation (94). These results are consistent with previous studies indicating that supportive neighborhood environments, including accessible recreational spaces, favorable environmental quality, and socially supportive activity cultures, are positively related to physical activity engagement among aging populations (95). From the perspective of the ecological model of health behavior, these findings suggest that older adults’ physical activity should be understood as the result of interactions between individual characteristics and community-level environmental contexts, rather than as a behavior determined solely by personal motivation or health awareness.

A key contribution of the present study lies in the simultaneous examination of both the overall community physical activity environment and its dimension-specific components (13). While previous studies have frequently relied on aggregated environmental indices, the present findings suggest that different environmental dimensions may show different patterns of association with physical activity levels (96). In particular, the natural environment and CUL showed comparatively stronger positive associations with PALE. However, the coefficients for FAC and SER should be interpreted cautiously. Although both FAC and SER were positively correlated with PALE in the bivariate analysis, their coefficients became non-significant or negative after simultaneous adjustment for other environmental dimensions. This pattern should not be interpreted as evidence that facilities or public services are unimportant. Rather, it may reflect conceptual overlap, shared explanatory variance, or possible suppression effects among closely related environmental dimensions. Therefore, the results suggest that community physical activity environments should be understood as an integrated system in which facilities, services, natural conditions, and activity culture may jointly shape behavioral opportunities and motivations (97–99).

The stronger association of the natural environment observed in this study aligns with research indicating that access to green spaces (100), safe walking environments and esthetically pleasing surroundings (101) enhances both psychological well-being and physical activity participation among older adults. Environmental comfort, perceived safety, and reduced environmental stressors may lower the perceived physical and psychological costs of engaging in outdoor activities, particularly for individuals experiencing age-related functional limitations. Consequently, improvements in environmental quality may operate through both direct accessibility mechanisms and indirect motivational pathways, strengthening behavioral adoption and maintenance (102).

Equally noteworthy is the prominent role of CUL identified in the present analysis. Social participation norms, peer encouragement, and collective engagement opportunities appear to play an important role in sustaining physical activity behaviors among older adults (63). Previous research has demonstrated that social cohesion and neighborhood-level behavioral norms can strongly influence health-related behaviors through mechanisms such as observational learning, normative reinforcement, and collective participation dynamics (103). The present findings reinforce these perspectives by suggesting that the cultural and social climate surrounding physical activity may represent a critical environmental mechanism through which communities promote sustained behavioral engagement (104). This observation highlights the importance of shifting public health strategies from infrastructure-only approaches toward integrated “environment–culture” interventions that combine physical improvements with community-based participation programs.

Importantly, the overall community physical activity environment remained positively associated with PALE after adjustment for lifestyle covariates, suggesting that the observed association was not fully explained by smoking frequency, alcohol consumption frequency, high-fat food consumption frequency, or physical activity frequency (105). Nevertheless, this result should be interpreted as an adjusted association rather than evidence of a causal environmental effect. In particular, PAF was included as a lifestyle covariate reflecting general activity frequency, whereas PALE was derived from the multi-domain weighted PASE total score. This distinction is important because both variables are related to physical activity but represent different measurement constructs. From a policy perspective, the findings suggest that improving neighborhood walkability, enhancing green infrastructure, and fostering supportive community participation climates may be relevant strategies for promoting physical activity among older adults (11, 106).

Beyond confirming established ecological theories, the present study offers an important contextual contribution by examining environmental determinants of physical activity within an urban community setting characterized by substantial environmental heterogeneity (11). Rapid urban development often produces uneven distributions of recreational resources, environmental quality, and community-level support systems, which may lead to disparities in opportunities for physical activity participation (81). The present findings suggest that improving both physical and socio-cultural environmental conditions at the community level may represent an effective strategy for reducing inequalities in physical activity engagement among older adults (9). Such integrated environmental approaches may be particularly relevant in aging societies, where promoting active lifestyles is increasingly recognized as a critical component of public health and long-term healthcare sustainability (107).

Overall, the results highlight the need for comprehensive community-based strategies that integrate environmental planning, community engagement initiatives, and health promotion programs (108). Rather than focusing exclusively on facility provision, effective public health interventions should emphasize the combined enhancement of environmental quality, accessibility, and supportive social-cultural environments to create sustainable activity-friendly communities for older adults (109).

## 6 Conclusion

This study examined the relationship between community physical activity environments and physical activity levels among older adults using a hierarchical analytical framework that accounted for demographic and lifestyle factors (90). The findings indicate that community environmental characteristics were significantly associated with physical activity participation in later life (44), with the inclusion of environmental indicators improving the explanatory power of the regression models. In particular, the natural environment and CUL showed comparatively stronger positive associations with PALE, highlighting the relevance of both environmental quality and socially supportive activity climates for older adults’ physical activity participation (85).

From a theoretical perspective, the results provide empirical support for ecological models of health behavior by showing that environmental contexts are associated with physical activity beyond individual demographic and lifestyle characteristics (32). The findings further suggest that community physical activity environments are multidimensional, with natural conditions, facilities, public services, and socio-cultural activity climates jointly forming the broader environmental context of physical activity. However, because these dimensions were highly inter-correlated, the dimension-specific coefficients should be interpreted as unique adjusted associations rather than isolated environmental effects. By distinguishing overall and dimension-specific environmental associations, the study advances current research by offering a more cautious and nuanced understanding of how community environments are related to active aging.

From a policy and public health perspective, the results underscore the importance of integrated community-level strategies aimed at creating activity-supportive environments for aging populations (110). Enhancing neighborhood green spaces, improving environmental accessibility and safety, and fostering supportive community participation cultures may collectively promote sustained physical activity engagement among older adults (111). Such environmental approaches complement traditional individual-level health promotion programs by shaping behavioral opportunities at the population level and may therefore represent a sustainable pathway for improving health outcomes in aging societies.

Future research should extend the present findings by employing longitudinal and multi-level study designs to examine causal mechanisms and dynamic interactions between environmental characteristics and behavioral processes over time. In addition, the integration of objective environmental indicators alongside perceived environmental measures may further strengthen understanding of the pathways through which community environments influence physical activity behavior (67). Collectively, continued research in this area will contribute to the development of evidence-based environmental and policy interventions designed to support healthy and active aging (112).

### 6.1 Limitations

Several limitations should be considered when interpreting the findings of the present study. First, the cross-sectional design limits the ability to establish causal relationships between community physical activity environments and physical activity levels among older adults. Although the hierarchical analytical framework allows for the examination of independent associations after controlling for demographic and behavioral factors, longitudinal and prospective studies are needed to confirm causal pathways and to examine how environmental changes over time influence behavioral outcomes.

Second, the study relied primarily on self-reported questionnaire data collected from the same respondents at a single time point. This may introduce recall bias, social desirability bias, common method variance, and same-source bias. In particular, respondents who reported more favorable community environments may also have been more likely to report higher levels of physical activity, which could inflate the observed associations. Although anonymous responses, confidentiality assurances, standardized instructions, and separated questionnaire sections were used to reduce this risk, common method and same-source bias cannot be fully excluded. Future research incorporating wearable device-based activity monitoring, administrative behavioral records, or repeated measurements could provide complementary evidence and strengthen measurement precision.

Third, the assessment of the community physical activity environment was based on respondents’ perceived environmental evaluations rather than objective geographic or spatial indicators. Perceived environmental measures capture individuals’ subjective experiences and behavioral interpretations of their surroundings, which are important predictors of behavioral engagement; however, they may not fully reflect objective environmental conditions. Integrating objective environmental assessments, such as geographic information system (GIS)-based environmental indicators, would allow for a more comprehensive evaluation of environmental influences.

Finally, although the analyses adjusted for several key demographic and lifestyle variables, the possibility of residual confounding cannot be entirely excluded. Unmeasured factors, such as health status, mobility limitations, or psychosocial characteristics, may also influence physical activity participation and partially account for observed associations. Despite these limitations, the study provides useful evidence on the association between community environmental factors and physical activity participation among older adults, offering insights for the development of environmental and community-level public health interventions aimed at promoting active aging.

## Data Availability

The raw data supporting the conclusions of this article will be made available by the authors, without undue reservation.

## References

[1] Gianfredi V Nucci D Pennisi F Maggi S Veronese N Soysal P. Aging, longevity, and healthy aging: the public health approach. Aging Clin Exp Res. (2025) 37:125. doi: 10.1007/s40520-025-03021-8, 40244306 PMC12006278

[2] Eckstrom E Neukam S Kalin L Wright J. Physical activity and healthy aging. Clin Geriatr Med. (2020) 36:671–83. doi: 10.1016/j.cger.2020.06.009, 33010902

[3] Dogra S Dunstan DW Sugiyama T Stathi A Gardiner PA Owen N. Active aging and public health: evidence, implications, and opportunities. Annu Rev Public Health. (2022) 43:439–59. doi: 10.1146/annurev-publhealth-052620-091107, 34910580

[4] Brach M De Bruin ED Levin O Hinrichs T Zijlstra W Netz Y. Evidence-based yet still challenging! Research on physical activity in old age. Eur Rev Aging Phys Act. (2023) 20:7. doi: 10.1186/s11556-023-00318-3, 36932320 PMC10022149

[5] Knight RL McNarry MA Sheeran L Runacres AW Thatcher R Shelley J . Moving forward: understanding correlates of physical activity and sedentary behaviour during COVID-19—an integrative review and socioecological approach. Int J Environ Res Public Health. (2021) 18:10910. doi: 10.3390/ijerph182010910, 34682653 PMC8535281

[6] Nakkeeran N Sacks E Srinivas PN Juneja A Gaitonde R Garimella S . Beyond behaviour as individual choice: a call to expand understandings around social science in health research. Wellcome Open Res. (2021) 6:212. doi: 10.12688/wellcomeopenres.17149.1, 34622015 PMC8453310

[7] Sarni ROS Kochi C Suano-Souza FI. Childhood obesity: an ecological perspective. J Pediatr. (2022) 98:S38–46. doi: 10.1016/j.jped.2021.10.002, 34780713 PMC9510906

[8] Lee Y Park S. Understanding of physical activity in social ecological perspective: application of multilevel model. Front Psychol. (2021) 12:622929. doi: 10.3389/fpsyg.2021.622929, 33746840 PMC7973361

[9] Zhang X Warner ME. Linking urban planning, community environment, and physical activity: a socio-ecological approach. Int J Environ Res Public Health. (2023) 20:2944. doi: 10.3390/ijerph20042944, 36833640 PMC9956976

[10] Peters M Muellmann S Christianson L Stalling I Bammann K Drell C . Measuring the association of objective and perceived neighborhood environment with physical activity in older adults: challenges and implications from a systematic review. Int J Health Geogr. (2020) 19:47. doi: 10.1186/s12942-020-00243-z, 33168094 PMC7654613

[11] Høyer-Kruse J Schmidt EB Hansen AF Pedersen MRL. The interplay between social environment and opportunities for physical activity within the built environment: a scoping review. BMC Public Health. (2024) 24:2361. doi: 10.1186/s12889-024-19733-x, 39215315 PMC11363614

[12] Stevens SM Joy MK Abrahamse W Milfont TL Petherick LM. Composite environmental indices—a case of rickety rankings. PeerJ. (2023) 11:e16325. doi: 10.7717/peerj.16325, 38099306 PMC10720475

[13] Salgado M Madureira J Mendes AS Torres A Teixeira JP Oliveira MD. Environmental determinants of population health in urban settings. A systematic review. BMC Public Health. (2020) 20:853. doi: 10.1186/s12889-020-08905-0, 32493328 PMC7271472

[14] Leese CJ Al-Zubaidi H Smith BH. Delivery of interventions for multiple lifestyle factors in primary healthcare settings: a narrative review addressing strategies for effective implementation. Lifestyle Med. (2024) 5:e110. doi: 10.1002/lim2.110

[15] Seyedrezaei M Becerik-Gerber B Awada M Contreras S Boeing G. Equity in the built environment: a systematic review. Build Environ. (2023) 245:110827. doi: 10.1016/j.buildenv.2023.110827

[16] Meredith SJ Cox NJ Ibrahim K Higson J McNiff J Mitchell S . Factors that influence older adults’ participation in physical activity: a systematic review of qualitative studies. Age Ageing. (2023) 52:afad145. doi: 10.1093/ageing/afad145, 37595070 PMC10438214

[17] Narsakka N Suhonen R Kielo-Viljamaa E Stolt M. Physical, social, and symbolic environment related to physical activity of older individuals in long-term care: a mixed-method systematic review. Int J Nurs Stud. (2022) 135:104350. doi: 10.1016/j.ijnurstu.2022.104350, 36116327

[18] Noh W Kim KY. Review of ecological approach factors affecting physical activity among older people. West J Nurs Res. (2022) 44:799–808. doi: 10.1177/01939459211017530, 34032167

[19] Li Y Yatsuya H Hanibuchi T Ota A Naito H Otsuka R . Positive association of physical activity with both objective and perceived measures of the neighborhood environment among older adults: the Aichi workers’ cohort study. Int. J. Environ. Res. Public Health. (2020) 17:7971. doi: 10.3390/ijerph17217971, 33138333 PMC7663542

[20] Duro CD Musa J Kayode T Singh M Rodriguez R. Promoting healthy aging: a review of community-based interventions to support active aging and independence. Int J Sci Res Arch. (2025) 16:74–83. doi: 10.30574/ijsra.2025.16.2.2281

[21] Catissi G Gouveia G Savieto RM Silva CPR de Almeida RS Borba GB . Nature-based interventions targeting elderly people’s health and well-being: an evidence map. Int J Environ Res Public Health. (2024) 21:112. doi: 10.3390/ijerph21010112, 38276806 PMC10815627

[22] Roberto CA. An inconvenient truth: difficult problems rarely have easy solutions. Behav Brain Sci. (2023) 46:e173. doi: 10.1017/S0140525X23000882, 37646295

[23] Arakawa Martins B Taylor D Barrie H Lange J Sok Fun Kho K Visvanathan R. Objective and subjective measures of the neighbourhood environment: associations with frailty levels. Arch Gerontol Geriatr. (2021) 92:104257. doi: 10.1016/j.archger.2020.104257, 32979550

[24] Ye X Du J Han Y Newman G Retchless D Zou L . Developing human-centered urban digital twins for community infrastructure resilience: a research agenda. J Plan Lit. (2023) 38:187–99. doi: 10.1177/08854122221137861, 37153810 PMC10162701

[25] McShane I Coffey B. Rethinking community hubs: community facilities as critical infrastructure. Curr Opin Environ Sustain. (2022) 54:101149. doi: 10.1016/j.cosust.2022.101149

[26] Igalla M Edelenbos J Van Meerkerk I. Institutionalization or interaction: which organizational factors help community-based initiatives acquire government support? Public Adm. (2021) 99:803–31. doi: 10.1111/padm.12728

[27] Segundo-Ortin M. Socio-cultural norms in ecological psychology: the education of intention. Phenom Cogn Sci. (2024) 23:1–19. doi: 10.1007/s11097-022-09807-9

[28] Zeng E Dong Y Yan L Lin A. Perceived safety in the neighborhood: exploring the role of built environment, social factors, physical activity and multiple pathways of influence. Buildings. (2022) 13:2. doi: 10.3390/buildings13010002

[29] Zhang X Gao X Wu D Xu Z Wang H. The role of big data in aging and older people’s health research: a systematic review and ecological framework. Sustainability. (2021) 13:11587. doi: 10.3390/su132111587

[30] Kumar S Underwood SH Masters JL Manley NA Konstantzos I Lau J . Ten questions concerning smart and healthy built environments for older adults. Build Environ. (2023) 244:110720. doi: 10.1016/j.buildenv.2023.110720

[31] Freiberger E Sieber CC Kob R. Mobility in older community-dwelling persons: a narrative review. Front Physiol. (2020) 11:881. doi: 10.3389/fphys.2020.00881, 33041836 PMC7522521

[32] Giles LV Koehle MS Saelens BE Sbihi H Carlsten C. When physical activity meets the physical environment: precision health insights from the intersection. Environ Health Prev Med. (2021) 26:68. doi: 10.1186/s12199-021-00990-w, 34193051 PMC8247190

[33] Chang P-J. Effects of the built and social features of urban greenways on the outdoor activity of older adults. Landsc Urban Plan. (2020) 204:103929. doi: 10.1016/j.landurbplan.2020.103929

[34] Pérez-Tejera F Anguera MT Guàrdia-Olmos J Dalmau-Bueno A Valera S. Examining perceived safety and park use in public open spaces: the case of Barcelona. J Environ Psychol. (2022) 81:101823. doi: 10.1016/j.jenvp.2022.101823

[35] Kurtenbach S. Neighbourhoods and social cohesion: why neighbourhoods still matter. Built Environ. (2024) 50:73–94. doi: 10.2148/benv.50.1.73

[36] Hailey V Bloomberg M Hamer M Fisher A. Association between neighbourhood cohesion and physical activity trajectories during the COVID-19 pandemic using data from understanding society: the UK household longitudinal study & COVID-19 sub-study. Prev Med Rep. (2023) 35:102392. doi: 10.1016/j.pmedr.2023.102392, 37680857 PMC10480663

[37] Dėdelė A Chebotarova Y Miškinytė A. Motivations and barriers towards optimal physical activity level: a community-based assessment of 28 EU countries. Prev Med. (2022) 164:107336. doi: 10.1016/j.ypmed.2022.107336 36334682

[38] Lee S Lee C An J. Psycho-social correlates of leisure-time physical activity (LTPA) among older adults: a multivariate analysis. Eur Rev Aging Phys Act. (2020) 17:6. doi: 10.1186/s11556-020-00238-6, 32158506 PMC7057464

[39] Manzi F Del Riccio M Naldi E Setola N Dellisanti C Lorini C . Elements of the built environment that can promote physical activity in elderly: an umbrella review. Eur J Pub Health. (2020) 30:ckaa165.236. doi: 10.1093/eurpub/ckaa165.236

[40] Sallis JF Cerin E Kerr J Adams MA Sugiyama T Christiansen LB . Built environment, physical activity, and obesity: findings from the international physical activity and environment network (IPEN) adult study. Annu Rev Public Health. (2020) 41:119–39. doi: 10.1146/annurev-publhealth-040218-043657, 32237990

[41] Bonaccorsi G Manzi F Del Riccio M Setola N Naldi E Milani C . Impact of the built environment and the neighborhood in promoting the physical activity and the healthy aging in older people: an umbrella review. Int J Environ Res Public Health. (2020) 17:6127. doi: 10.3390/ijerph17176127, 32842526 PMC7504170

[42] Abu-Omar K Gelius P Messing S. Physical activity promotion in the age of climate change. F1000Res. (2020) 9:349. doi: 10.12688/f1000research.23764.2, 33628429 PMC7883311

[43] Schweiker M Ampatzi E Andargie MS Andersen RK Azar E Barthelmes VM . Review of multi-domain approaches to indoor environmental perception and behaviour. Build Environ. (2020) 176:106804. doi: 10.1016/j.buildenv.2020.106804

[44] Peterman JE Loy S Carlos J Arena R Kaminsky LA. Increasing physical activity in the community setting. Prog Cardiovasc Dis. (2021) 64:27–32. doi: 10.1016/j.pcad.2020.10.008, 33130191

[45] Duarte G Okan Y Johnston M Ortiz L Villalobos Dintrans P Armayones M. Introduction to behavioral science and its practical applications in public health. Medwave. (2025) 25:e3017–7. doi: 10.5867/medwave.2025.01.3017, 39836869

[46] Rigby BP Dodd-Reynolds CJ Oliver EJ. The understanding, application and influence of complexity in national physical activity policy-making. Health Res Policy Sys. (2022) 20:59. doi: 10.1186/s12961-022-00864-9, 35641991 PMC9153223

[47] Magriplis E Bakogianni I Arvaniti F Kotopoulou S Smiliotopoulos T Karatzi K . Addressing the importance of recreational area availability on physical activity level: results from the Hellenic National Nutrition and Health Survey (HNNHS). J Public Health. (2025). doi: 10.1007/s10389-025-02441-0

[48] Zhu X Ory MG Xu M Towne SD Jr Lu Z Hammond T . Physical activity impacts of an activity-friendly community: a natural experiment study protocol. Front Public Health. (2022) 10:929331. doi: 10.3389/fpubh.2022.929331, 35784244 PMC9240399

[49] Jenkins M Lee C Houge Mackenzie S Hargreaves EA Hodge K Calverley J. Nature-based physical activity and hedonic and eudaimonic wellbeing: the mediating roles of motivational quality and nature relatedness. Front Psychol. (2022) 13:783840. doi: 10.3389/fpsyg.2022.783840, 35153952 PMC8830485

[50] Kilgour AHM Rutherford M Higson J Meredith SJ McNiff J Mitchell S . Barriers and motivators to undertaking physical activity in adults over 70—a systematic review of the quantitative literature. Age Ageing. (2024) 53:afae080. doi: 10.1093/ageing/afae080, 38651329 PMC11036106

[51] Li D Xu H Kang Y Steemers K. Systematic review: landscape characteristics correlated with physical activity of the elderly people. Land (Basel). (2023) 12:605. doi: 10.3390/land12030605

[52] Soga M Gaston KJ. Do people who experience more nature act more to protect it? A meta-analysis. Biol Conserv. (2024) 289:110417. doi: 10.1016/j.biocon.2023.110417

[53] Pedersen MRL Bredahl TVG Elmose-Østerlund K Hansen AF. Motives and barriers related to physical activity within different types of built environments: implications for health promotion. Int J Environ Res Public Health. (2022) 19:9000. doi: 10.3390/ijerph19159000, 35897374 PMC9330905

[54] Levinger P Dreher BL Soh S-E Dow B Batchelor F Hill KD. Results from the ENJOY MAP for HEALTH: a quasi experiment evaluating the impact of age-friendly outdoor exercise equipment to increase older people’s park visitations and physical activity. BMC Public Health. (2024) 24:1663. doi: 10.1186/s12889-024-19042-3, 38909183 PMC11193282

[55] Hu D Zhou S Crowley-McHattan ZJ Liu Z. Factors that influence participation in physical activity in school-aged children and adolescents: a systematic review from the social ecological model perspective. Int J Environ Res Public Health. (2021) 18:3147. doi: 10.3390/ijerph18063147 33803733 PMC8003258

[56] Chen Y Shah S Chen Y Owen AJ Ekegren CL Ilic D . Barriers to and facilitators of physical activity among community-dwelling older adults: a systematic review. BMJ Open. (2025) 15:e095260. doi: 10.1136/bmjopen-2024-095260, 40854831 PMC12382567

[57] Bonnell K Michalovic E Koch J Pagé V Ramsay J Gainforth HL . Physical activity for individuals living with a physical disability in Quebec: issues and opportunities of access. Disabil Health J. (2021) 14:101089. doi: 10.1016/j.dhjo.2021.101089, 33722579

[58] Franke T Sims-Gould J Nettlefold L Ottoni C McKay HA. “It makes me feel not so alone”: features of the choose to move physical activity intervention that reduce loneliness in older adults. BMC Public Health. (2021) 21:312. doi: 10.1186/s12889-021-10363-1, 33549090 PMC7865112

[59] Troutman-Jordan M O’Brien T Keaton M. Older adults’ views and attitudes on physical activity; reasons to participate and abstain. J Community Health Nurs. (2021) 38:232–43. doi: 10.1080/07370016.2021.1972247 34787041

[60] Killingback C Tsofliou F Clark C. “Everyone’s so kind and jolly it boosts my spirits, if you know what I mean”: a humanising perspective on exercise programme participation. Scand J Caring Sci. (2022) 36:162–72. doi: 10.1111/scs.12973, 33719077

[61] Singh KN Sendall MC Crane P. Understanding sociocultural influences on physical activity in relation to overweight and obesity in a rural indigenous community of Fiji islands. J Racial Ethnic Health Disparit. (2023) 10:1508–17. doi: 10.1007/s40615-022-01336-0, 35676494 PMC10163082

[62] Fischer R Karl JA. Predicting behavioral intentions to prevent or mitigate COVID-19: a cross-cultural meta-analysis of attitudes, norms, and perceived behavioral control effects. Soc Psychol Personal Sci. (2022) 13:264–76. doi: 10.1177/19485506211019844

[63] Park J-H Prochnow T Vigil JA Smith ML. A systematic literature review of the relationships between social and interpersonal factors and physical activity among older adults. Am J Health Promot. (2025) 39:664–78. doi: 10.1177/08901171241302925, 39575876 PMC12014959

[64] Maghsoodi Tilaki MJ Farhad S. Revitalising historic fabrics: the influence of identity and community belonging. Geogr Res. (2025) 63:573–93. doi: 10.1111/1745-5871.70018

[65] Caperon L Saville F Ahern S. Developing a socio-ecological model for community engagement in a health programme in an underserved urban area. PLoS One. (2022) 17:e0275092. doi: 10.1371/journal.pone.0275092, 36155664 PMC9512167

[66] Preissner CE Charles K Knäuper B Kaushal N. Predicting decisional determinants of physical activity among older adults: an integrated behavior approach. J Aging Health. (2022) 34:569–80. doi: 10.1177/08982643211049079, 34657497 PMC9446452

[67] Menardo E De Dominicis S Pasini M. Exploring perceived and objective measures of the neighborhood environment and associations with physical activity among adults: a review and a meta-analytic structural equation model. Int J Environ Res Public Health. (2022) 19:2575. doi: 10.3390/ijerph19052575, 35270267 PMC8909183

[68] Grootens-Wiegers P Van Den Eynde E Halberstadt J Seidell JC Dedding C. The ‘stages towards completion model’: what helps and hinders children with overweight or obesity and their parents to be guided towards, adhere to and complete a group lifestyle intervention. Int J Qual Stud Health Well Being. (2020) 15:1735093. doi: 10.1080/17482631.2020.1735093, 32148191 PMC7144242

[69] Gooderham GK Handy TC. Metacognitive function in young adults is impacted by physical activity, diet, and sleep patterns. PLoS One. (2025) 20:e0317253. doi: 10.1371/journal.pone.0317253, 39787158 PMC11717208

[70] Gidney G Bocarro JN Bunds K Koenigstorfer J. The relationship between the environment and physical activity-related motivational trajectories. Psychol Sport Exerc. (2024) 75:102719. doi: 10.1016/j.psychsport.2024.102719, 39182749

[71] Zhang H Shi X Wang K Xue J Song L Sun Y. Intertemporal lifestyle changes and carbon emissions: evidence from a China household survey. Energy Econ. (2020) 86:104655. doi: 10.1016/j.eneco.2019.104655

[72] Nurchis MC Di Pumpo M Perilli A Greco G Damiani G. Nudging interventions on alcohol and tobacco consumption in adults: a scoping review of the literature. Int J Environ Res Public Health. (2023) 20:1675. doi: 10.3390/ijerph20031675, 36767077 PMC9913966

[73] Bahrami H Tafrihi M. Global trends of cancer: the role of diet, lifestyle, and environmental factors. Cancer Innov. (2023) 2:290–301. doi: 10.1002/cai2.76, 38089751 PMC10686168

[74] Kobayashi T Tani Y Kino S Fujiwara T Kondo K Kawachi I. Prospective study of engagement in leisure activities and all-cause mortality among older Japanese adults. J Epidemiol. (2022) 32:245–53. doi: 10.2188/jea.JE20200427, 33551388 PMC9086310

[75] Chang S Chien N Pui-Man Wai J Chiang C Yu C. Examining the links between regular leisure-time physical activity, sitting time and prefrailty in community-dwelling older adults. J Adv Nurs. (2021) 77:2761–73. doi: 10.1111/jan.14807 33619783

[76] Aira T Vasankari T Heinonen OJ Korpelainen R Kotkajuuri J Parkkari J . Psychosocial and health behavioural characteristics of longitudinal physical activity patterns: a cohort study from adolescence to young adulthood. BMC Public Health. (2023) 23:2156. doi: 10.1186/s12889-023-17122-4, 37924075 PMC10625285

[77] Breland JY Wong JJ McAndrew LM. Are common sense model constructs and self-efficacy simultaneously correlated with self-management behaviors and health outcomes: a systematic review. Health Psychol Open. (2020) 7:2055102919898846. doi: 10.1177/2055102919898846, 32030192 PMC6978827

[78] Marashi MY Nicholson E Ogrodnik M Fenesi B Heisz JJ. A mental health paradox: mental health was both a motivator and barrier to physical activity during the COVID-19 pandemic. PLoS One. (2021) 16:e0239244. doi: 10.1371/journal.pone.0239244, 33793550 PMC8016471

[79] Pedersen MRL Hansen AF Elmose-Østerlund K. Motives and barriers related to physical activity and sport across social backgrounds: implications for health promotion. Int J Environ Res Public Health. (2021) 18:5810. doi: 10.3390/ijerph18115810, 34071630 PMC8198157

[80] Besser LM Brenowitz WD Meyer OL Hoermann S Renne J. Methods to address self-selection and reverse causation in studies of neighborhood environments and brain health. Int J Environ Res Public Health. (2021) 18:6484. doi: 10.3390/ijerph18126484, 34208454 PMC8296350

[81] Moreno-Llamas A García-Mayor J De La Cruz-Sánchez E. Urban–rural differences in perceived environmental opportunities for physical activity: a 2002–2017 time-trend analysis in Europe. Health Promot Int. (2023) 38:daad087. doi: 10.1093/heapro/daad087, 37540055

[82] Mutz J Roscoe CJ Lewis CM. Exploring health in the UK biobank: associations with sociodemographic characteristics, psychosocial factors, lifestyle and environmental exposures. BMC Med. (2021) 19:240. doi: 10.1186/s12916-021-02097-z, 34629060 PMC8504077

[83] Parkinson JA Gould A Knowles N West J Goodman AM. Integrating systems thinking and Behavioural science. Behav Sci. (2025) 15:403. doi: 10.3390/bs15040403, 40282025 PMC12023936

[84] Akimova ET Breen R Brazel DM Mills MC. Gene-environment dependencies lead to collider bias in models with polygenic scores. Sci Rep. (2021) 11:9457. doi: 10.1038/s41598-021-89020-x, 33947934 PMC8097011

[85] Meredith SJ Cox N Ibrahim K Higson J McNiff J Mitchell . 979 factors that influence older adults’ participation in physical activity: a systematic review of qualitative studies. Age Ageing. (2022) 51:afac125.002. doi: 10.1093/ageing/afac125.002 PMC10438214 37595070

[86] Azizan A Fadzil NHM. What stops us and what motivates us? A scoping review and bibliometric analysis of barriers and facilitators to physical activity. Ageing Res Rev. (2024) 99:102384. doi: 10.1016/j.arr.2024.102384, 38914263

[87] Brown CEB Richardson K Halil-Pizzirani B Atkins L Yücel M Segrave RA. Key influences on university students’ physical activity: a systematic review using the theoretical domains framework and the COM-B model of human behaviour. BMC Public Health. (2024) 24:418. doi: 10.1186/s12889-023-17621-4, 38336748 PMC10854129

[88] Kendal D Egerer M Byrne JA Jones PJ Marsh P Threlfall CG . City-size bias in knowledge on the effects of urban nature on people and biodiversity. Environ Res Lett. (2020) 15:124035. doi: 10.1088/1748-9326/abc5e4

[89] Lee Y-H Fan S-Y. Psychosocial and environmental factors related to physical activity in middle-aged and older adults. Sci Rep. (2023) 13:7788. doi: 10.1038/s41598-023-35044-4, 37179430 PMC10182976

[90] Zhang J Bloom I Dennison EM Ward KA Robinson SM Barker M . Understanding influences on physical activity participation by older adults: a qualitative study of community-dwelling older adults from the Hertfordshire cohort study, UK. PLoS One. (2022) 17:e0263050. doi: 10.1371/journal.pone.0263050, 35077522 PMC8789143

[91] Andersson S Svensson G Molina-Castillo F Otero‐Neira C Lindgren J Karlsson NPE . Sustainable development—direct and indirect effects between economic, social, and environmental dimensions in business practices. Corp Soc Responsib Environ Manag. (2022) 29:1158–72. doi: 10.1002/csr.2261

[92] Jiang J Xia Z Sun X Wang X Luo S. Social infrastructure and street networks as critical infrastructure for aging friendly community design: mediating the effect of physical activity. Int. J. Environ. Res. Public Health. (2022) 19:11842. doi: 10.3390/ijerph191911842, 36231144 PMC9565500

[93] Davids K Otte F Rothwell M. Adopting an ecological perspective on skill performance and learning in sport. European J. Human Mov. (2021) 46:667. doi: 10.21134/eurjhm.2021.46.667

[94] Zhang F Loo BPY Wang B. Aging in place: from the neighborhood environment, sense of community, to life satisfaction. Ann Am Assoc Geogr. (2022) 112:1484–99. doi: 10.1080/24694452.2021.1985954

[95] Liu Z Kemperman A Timmermans H. Influence of neighborhood characteristics on physical activity, health, and quality of life of older adults: a path analysis. Front Public Health. (2021) 9:783510. doi: 10.3389/fpubh.2021.783510, 34900922 PMC8652252

[96] Zhang C Shi D Xiao Z. Integrating variable importance and spatial heterogeneity to reveal the environmental effects on outdoor jogging. ComputUrban Sci. (2024) 4:45. doi: 10.1007/s43762-024-00158-6, 30311153

[97] Keskinen KE Gao Y Rantakokko M Rantanen T Portegijs E. Associations of environmental features with outdoor physical activity on weekdays and weekend days: a cross-sectional study among older people. Front Public Health. (2020) 8:578275. doi: 10.3389/fpubh.2020.578275, 33194978 PMC7661781

[98] Makahleh HY Taamneh MM Dissanayake D. Promoting sustainable transport: a systematic review of walking and cycling adoption using the COM-B model. Future Transp. (2025) 5:79. doi: 10.3390/futuretransp5030079

[99] Butson M Jeanes R O’Connor J. Experiences of older adults leisure-time physical activity in aquatic and leisure facilities. World Leis J. (2025) 67:124–44. doi: 10.1080/16078055.2024.2351077

[100] Nordh H Aamodt G Nordbø ECA. Greater perceived access to green spaces near homes: safer and more satisfied residents. J Environ Psychol. (2024) 96:102332. doi: 10.1016/j.jenvp.2024.102332

[101] Abildso CG Daily SM Meyer MRU Edwards MB Jacobs L McClendon M . Environmental factors associated with physical activity in rural U.S. counties. Int J Environ Res Public Health. (2021) 18:7688. doi: 10.3390/ijerph18147688, 34300138 PMC8307667

[102] Shafiei A Maleksaeidi H. Pro-environmental behavior of university students: application of protection motivation theory. Glob Ecol Conserv. (2020) 22:e00908. doi: 10.1016/j.gecco.2020.e00908

[103] Rosenblatt AM Crews DC Powe NR Zonderman AB Evans MK Tuot DS. Association between neighborhood social cohesion, awareness of chronic diseases, and participation in healthy behaviors in a community cohort. BMC Public Health. (2021) 21:1611. doi: 10.1186/s12889-021-11633-8, 34479522 PMC8414876

[104] Rio CJ Saligan LN. Understanding physical activity from a cultural-contextual lens. Front Public Health. (2023) 11:1223919. doi: 10.3389/fpubh.2023.1223919, 37601221 PMC10436229

[105] D’Amore C Saunders S Bhatnagar N Griffith LE Richardson J Beauchamp MK. Determinants of physical activity in community-dwelling older adults: an umbrella review. Int J Behav Nutr Phys Act. (2023) 20:135. doi: 10.1186/s12966-023-01528-9, 37990225 PMC10664504

[106] Benton JS French DP. Untapped potential of unobtrusive observation for studying health behaviors. JMIR Public Health Surveill. (2024) 10:e46638. doi: 10.2196/46638, 38381483 PMC10918536

[107] Cocchi C Zazzara MB Levati E Calvani R Onder G. How to promote healthy aging across the life cycle. Eur J Intern Med. (2025) 135:5–13. doi: 10.1016/j.ejim.2025.03.003, 40107887

[108] Stover J Avadhanula L Sood S. A review of strategies and levels of community engagement in strengths-based and needs-based health communication interventions. Front Public Health. (2024) 12:1231827. doi: 10.3389/fpubh.2024.1231827, 38655513 PMC11035763

[109] Chen S Bao Z Chen J Yang L Lou V. Sustainable built environment for facilitating public health of older adults: evidence from Hong Kong. Sustain Dev. (2022) 30:1086–98. doi: 10.1002/sd.2303

[110] Jackman PC Dargue-Fox EJ Hawkins RM Murphy N Clarke M Swann C . Physical activity in age-friendly cities and communities: a scoping review with recommendations to support policy and practice. BMC Public Health. (2025) 25:4107. doi: 10.1186/s12889-025-25108-7, 41286710 PMC12642209

[111] Ward M Gibney S O’Callaghan D Shannon S. Age-friendly environments, active lives? Associations between the local physical and social environment and physical activity among adults aged 55 and older in Ireland. J Aging Phys Act. (2020) 28:140–8. doi: 10.1123/japa.2019-0012, 31629358

[112] Lum TYS. The pathways to healthy ageing: evidence from longitudinal studies and real-time data. Innov Aging. (2021) 5:467–7. doi: 10.1093/geroni/igab046.1806

